# Microbial community composition and diversity in rice straw digestion bioreactors with and without dairy manure

**DOI:** 10.1007/s00253-018-9243-7

**Published:** 2018-07-27

**Authors:** A. M. Zealand, R. Mei, P. Papachristodoulou, A. P. Roskilly, W. T. Liu, David W. Graham

**Affiliations:** 1 School of Engineering, Newcastle University, Cassie Building, Newcastle upon Tyne, NE1 7RU UK; 2 Department of Civil and Environmental Engineering, University of Illinois at Urbana-Champaign, 205 North Mathews Ave, Urbana, IL 61801 USA; 3 Sir Joseph Swan Centre for Energy Research, Newcastle University, Newcastle upon Tyne, NE1 7RU UK

**Keywords:** 16S rDNA amplicon, Illumina sequencing, Anaerobic digestion, Rice straw, Feeding frequency, Organic loading rate, Methane yields

## Abstract

Anaerobic digestion (AD) uses a range of substrates to generate biogas, including energy crops such as globally abundant rice straw (RS). Unfortunately, RS is high in lignocellulosic material and has high to C:N ratios (~80:1), which makes it (alone) a comparatively poor substrate for AD. Co-digestion with dairy manure (DM) has been promoted as a method for balancing C:N ratios to improve RS AD whilst also treating another farm waste and co-producing a potentially useful fertiliser. However, past co-digestion studies have not directly compared RS AD microbial communities with and without DM additions, which has made it hard to assess all impacts of DM addition to RS AD processes. Here, four RS:DM ratios were contrasted in identical semi-continuous-fed AD bioreactors, and 100% RS was found to produce the highest specific methane yields (112 mL CH_4_/g VS/day; VS, volatile solids), which is over double yields achieved in the reactor with the highest DM content (30:70 RS:DM by mass; 48 mL CH_4_/g VS/day). To underpin these data, microbial communities were sequenced and characterised across the four reactors. Dominant operational taxonomic units (OTUs) in the 100% RS unit were *Bacteroidetes*/*Firmicutes*, whereas the 30:70 RS:DM unit was dominated by *Proteobacteria*/*Spirochaetes*, suggesting major microbial community shifts occur with DM additions. However, community richness was lowest with 100% RS (despite higher specific yields), suggesting particular OTUs may be more important to yields than microbial diversity. Further, ambient VFA and VS levels were significantly higher when no DM was added, suggesting DM-amended reactors may cope better with higher organic loading rates (OLR). Results show that RS AD without DM addition is feasible, although co-digestion with DM will probably allow higher OLRs, resulting in great RS throughput in farm AD units.

**Electronic supplementary material:**

The online version of this article (10.1007/s00253-018-9243-7) contains supplementary material, which is available to authorized users.

## Introduction

Anaerobic digestion (AD) uses a range of substrates to generate biogas, including food waste, energy crops, wastewater sludge and animal slurry. Lignocellulosic material, such as rice straw (RS), is not an ideal substrate as it is comparatively recalcitrant to AD and tends to generate lower biogas yields compared with many other crops (Mussoline et al. 2013). However, the demand for biogas in many parts of the world is increasing because the use of traditional fuels has many negative impacts, including increased deforestation (i.e. wood) and greater air pollution as particulate matter. Local biogas production, such as via RS AD, could reduce such pollution, offering a ‘cleaner’ alternative for lighting, heating and/or electricity generation (Krishania et al. 2013).

Various operating options exist for RS AD, but RS often has very high C:N ratios (Mussoline et al. 2013). High C:N ratios can result in incomplete digestion, whereas lower C:N ratios can lead to ammonia inhibition, making the targeted C:N ratio for AD roughly 25–30:1 (Monnet 2003; Ward et al. 2008). In low nitrogen systems, methanogens struggle to produce optimal biogas levels, whereas in lower C:N systems, excess nitrogen causes ammonia accumulation, pH increase, and inhibition (Monnet 2003). RS has a natural C:N ratio of up to 80:1 (Mussoline et al. 2013) and rebalancing C:N ratios to 25:1 can conditionally improves biogas yields (Lei et al. 2010). Co-digestion with manure is seen as a cost-effective method of balancing the C:N ratio in AD whilst using another large waste stream, potentially producing a rich fertiliser (Li et al. 2014a, 2015) and possibly increasing methane yields (Wang et al. 2014; Li et al. 2015). Manure co-digestion can add moisture and seed microbes to an AD system, potentially improving syntrophic potential and microbial growth (El-Mashad and Zhang 2010; Silvestre et al. 2013).

Therefore, co-digesting RS with manure has benefits, but the impact of manure on AD microbial communities related to specific methane yields and also pathogens in AD digestates is not known. Here, we assessed the effects of dairy manure (DM) co-digestion with RS in bench-scale bioreactors fed at different RS:DM ratios. Reactor performance was monitored, and microbial communities were characterised using 16S rDNA amplicon sequencing to assess how eubacteria and archaea composition and diversity vary as a function of DM additions and RS co-digestion conditions.

## Materials and methods

### Experimental conditions and analyses

Four 2.5 L reactors with control towers were used as the AD units, each with working volumes of 2.0 L. The glass airtight-sealed reactors consisted of a heating jacket set to 37 °C, a biogas sampling bag and a paddle stirrer. Overall, the reactors were operated for 150 days of which the first 75 days were used to acclimate the reactors to different RS:DM mixes. The same reactors had been operated for over 250 days prior to this experiment at different RS organic loading rates (OLRs); therefore, microbial communities were already acclimated to RS before DM amendments were commenced.

During acclimation with DM, the sludge inoculum was operated in draw-fill mode (digester sludge removal prior to feed addition) with a 25-day hydraulic retention time (HRT) and an OLR of 1.0 g VS/L/day (chosen based on previous assays reported in Zealand et al. (2017)) fed once every 7 days. After three HRTs, pH and VFA levels in the reactors had become consistent with time and the formal experiment was commenced (defined as time 0 here). Operationally, a feeding frequency (FF) of one in 7 days and an OLR of 1 g VS/L/day comprised 560 mL of reactor volume being removed per week, with 14 g VS RS (1.0 mm sieve) being added with 560 mL of distilled water. Four RS:DM ratios were assessed here at the same OLR (see Table 1). Reactors were named ‘RS100’, ‘RS90’, ‘RS70’ and ‘RS30’ based on the percentage of RS (by mass) in each reactor feed mixture.

**Table 1:** Rice straw and dairy manure feeding ratios for each AD reactor

Reactor	1	2	3	4
Rice straw (% of VS)	100	90	70	30
Dairy manure (% of VS)	0	10	30	70
C:N	60:1	40:1	24:1	13:1
Reactor code	RS100	RS90	RS70	RS30

### Analysis

Total solids (TS), volatile solids (VS; combustible solids at 550 °C ignition and presumed organic fraction), moisture content (MC), ash content (AC) and total C and N were analysed using American Public Health Association Standard Methods (APHA 1998). VFA analysis was performed three times per week and consisted of filtering each sample through a 0.2-μm PES syringe filter before mixing with 0.1 M octane sulphonic acid (1:1) and sonicating for 40 min. Once sonicated, samples were analysed using ion chromatography Dionex Aquion system equipped with an AS-AP auto sampler with Chameleon 7 Software. RS composition data are provided in Table 2.

**Table 2:** Overall mean total solids (TS), volatile solids (VS), moisture content (MC), ash content (AC) and total C and N levels

Parameter	Unit	Rice Straw	Dairy Manure
Total solids	% DW^a^	93.5 ± 0.1^b^	11.1 ± 0.3
Volatile solids	% DW	87.5 ± 0.2	84.5 ± 0.3
Moisture content	% DW	6.50 ± 0.1	89.7 ± 0.3
Ash content	% DW	12.5 ± 0.2	25.7 ± 0.7
C	% DW	40.1 ± 0.0	40.9 ± 0.4
N	% DW	0.66 ± 0.0	4.09 ± 0.0
C:N	Ratio	60.4	10.0
Calorific content	MJ/kg	14.7 ± 0.7	–^c^

^a^DW is an abbreviation of dry weight—the weight of sample at standard temperature and pressure

^b^Standard error (*n* = 3)

^c^No data for dairy manure calorific content

Daily biogas volume was determined using a 100-μL gas tight syringe (SGE, Australia). Biogas samples were collected at the same time of day and analysed immediately. To quantify methane content (% CH_4_), gas samples were directly injected into a Carlo Erba HRGC 5160 GC-FID fitted with a HP-PLOT Q column maintained at 35 °C with hydrogen as the carrier gas and Atlas software. A seven-point calibration was performed before and after each analysis session by injecting neat standards spanning expected methane concentrations (up to 80% CH_4_, Scientific Technical Gases, UK). All injections were made in triplicate and the standard calibration required a minimum *R*^2^ of 0.99. The volume of biogas was collected over time in 1.0 L Supel-Inert Multi-Layer Foil bags (Sigma Aldrich) before daily extraction using a 1 L gas tight syringe (SGE). Biogas and methane analysis was normalised to specific production (mL/g VS/day), corrected for moisture, standard temperature and pressure (STP) and headspace volume, according to the VDI Standard: 4630 (2006).

### Sample collection for sequencing and DNA preparation

Samples for DNA extraction (2 mL each) were collected in triplicate from the reactors after 0, 37 and 75 days of operations after acclimation. Raw DM samples also were collected in triplicate to compliment the reactor samples. Samples were always stored at − 20 °C before further processing. For each sample, DNA was extracted following the instructions of the FastDNA SPIN Kit for Soil (MP Biomedicals, Carlsbad, CA, USA). Extracted DNA was assessed for purity and quantified using a Qubit 2.0 Fluorometer (Invitrogen) to ensure adequate quantity and quality prior to sequencing.

### Sequencing analysis

Sequencing of extracted DNA (concentration of 1 and 10 ng/μL and a volume of 10–20 μL) was undertaken by LGC Genomics GmbH in Berlin, Germany. Analysis consisted of PCR amplification using universal forward primer (U341F) CCTAYGGGRBGCASCAG and universal reverse primer (U806R) GGACTACNNGGGTATCTAAT, targeting the V3-V4 16S DNA region (Klindworth et al. 2013). The standard protocol was 10 cycles of touchdown PCR (annealing 61–55 °C, decreasing 0.6 °C per cycle), before 26 standard 2-step PCR cycles at 55 °C. Quality control (agarose gel check), library preparation including tagging, equimolar mixing and clean-up were completed. 16S rDNA amplicon sequencing was then performed on Illumina MiSeq V3 (2 × 300 bp).

Bioinformatics analysis consisted of inline barcode demultiplexing, adaptor clipping and amplicon pre-processing using Mothur (Schloss et al. 2009): pair joining, filtering, alignment against Silva 16S, subsampling 5000–25,000 reads per sample, denoising and chimera removal. OTU picking used Mothur with clustering aligned sequences at 97% identity. Specific OTU analysis included assigning taxonomy on the Greengenes database (version 13_8). Predominant OTUs were defined as having ≥ 0.5% abundance in a sample. Phylogenetic analysis of predominant OTUs was performed with the ARB programme (Ludwig et al. 2004), using neighbour-joining and parsimony methods with 1000 bootstrap replication (McDonald et al. 2012; Kuroda et al. 2016). Additional information is provided in Supporting Information (SI).

### Data analyses

Analysis of variance (ANOVA) with Tukey comparison was used to compare mean CH_4_ content and biogas yields with significance defined as 95% confidence (*p* ≤ 0.05). All statistical analyses and figure plots were conducted or developed using Microsoft Excel and Minitab 17 (Leadtools Technologies Inc., version 17.1.0, 2014). PRIMER 7 (PRIMER-E, Plymouth, UK) was used to perform principal coordinates analysis (PCoA) and distance-based linear modelling (DistLM) of log-transformed, normalised, RS composition data.

Alpha diversity and beta diversity, based on weighted UniFrac distances, were calculated in QIIME 1.9.1. PRIMER 7 (PRIMER-E, Plymouth, UK) was used for principal component analysis (PCA), metric-multidimensional scaling (MDS), heat map development to visualise beta diversity, permutational analysis of variance (PERMANOVA) and for analysis of similarities (ANOSIM). To compare microbial diversity with performance data, RELATE, BEST and DistLM (distance-based linear model) were used for weighted UniFrac distances, employing Bray-Curtis distance (after square root transformation) (Ling et al. 2016; Mei et al. 2016).

Observed OTUs, Chao1, Simpson’s and Shannon’s Indexes were plotted and compared using ANOVA with Tukey comparison in Minitab 17 (Leadtools Technologies Inc., version 17.1.0, 2014). Group significant differences were compared in STAMP v2.1.3 using the *t* test. These methods were chosen to identify possible correlations/groupings and significant relationships at each point of analysis. The sequence data obtained in this study are deposited at NCBI GenBank with accession no. MG852175 - MG855654.

## Results

### Effect of dairy manure on reactor performance

**Fig. 1:**
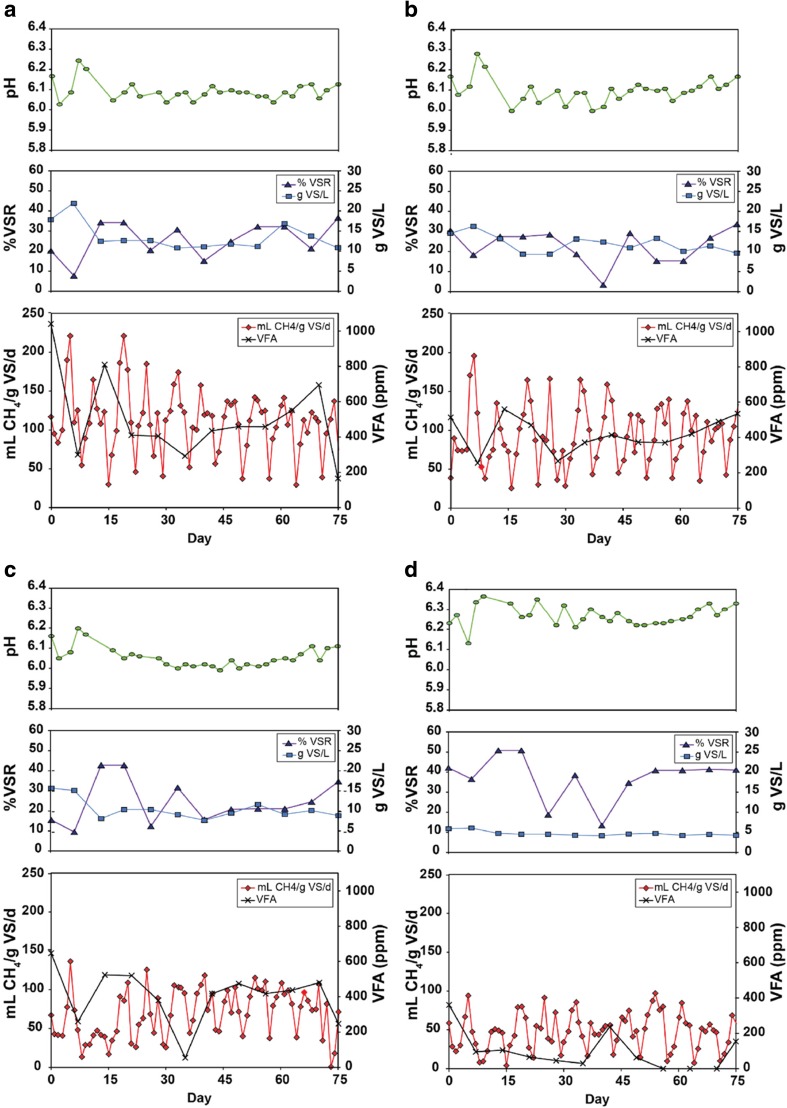
Time-course data of digester performance post-acclimation for pH, % VSR with g VS/L and methane yield with VFA concentration for **a** RS100, **b** RS90, **c** RS70 and **d** RS30

Stable bioreactor operations were confirmed before any samples were collected for microbial community characterisation. Stability was defined as when no statistically significant differences (using ANOVA) in biogas yields were apparent when comparing sequential HRTs of operating time. Biogas production ‘stability’ was achieved after the third HRT, after which specific gas yields, CH_4_ content, VS, pH and total VFA data were tallied and summarised (see Table 3). Time-course data (after acclimation) for pH, VS, CH_4_ yields and total VFA are shown in Fig. 1a–d (all time-course data is provided in Figure S1; see SI). Ammonia was always below detection limits throughout the experiment.

**Table 3:** Mean performance data after acclimation for AD reactors with different feeding regimes

Reactor	RS100	RS90	RS70	RS30
Biogas yield (mL/g VS/day)	*222*^a^ ± *5*.*2*^b^	193 ± 5.2	156 ± 4.3	115 ± 4.7
Methane content (% CH_4_)	*50*.*9* ± *1*.*7*	48.2 ± 1.5	43.2 ± 1.8	40.8 ± 1.6
Methane yield (mL CH_4_/g VS/day)	*112* ± *4*.*6*	94.2 ± 4.3	69.9 ± 3.5	47.5 ± 2.7
g VS/L	13.9 ± 1.0	11.9 ± 0.6	10.6 ± 0.7	*4*.*7* ± *0*.*2*
% VS reduction	26.3 ± 2.5	22.8 ± 2.5	24.7 ± 3.2	*37*.*5* ± *3*.*2*
pH	6.1 ± 0.01	6.1 ± 0.01	6.0 ± 0.01	6.3 ± 0.01
Total VFA (ppm^c^)	506 ± 69	420 ± 28	401 ± 44	*97* ± *31*
Formic acid (ppm)	11.6 ± 9.7	15.3 ± 12	16.8 ± 15	18.0 ± 10
Acetic acid (ppm)	145 ± 32	118 ± 15	116 ± 21	106 ± 37
Propionic acid (ppm)	319 ± 50	259 ± 20	256 ± 32	*36*.*9* ± *12*
Isobutyric acid (ppm)	51.9 ± 21	57.0 ± 26	64.5 ± 32	64.2 ± 33

^a^Italic indicates the significance (*p* < 0.05) for that parameter on each row of data, i.e. highest gas/VSR, lowest VS/acid

^b^Standard error (For OLR 1.0 g VS/L/day *n* = 76 for biogas and methane, *n* = 12 for VS and total VFA, *n* = 30 for pH and *n* = 3–12 for individual VFAs

^c^Synonymous with mg/L and is the traditional units used in with practical AD systems

Mean specific biogas yields ranged from ~115 to 222 mL/g VS/day across reactors, whereas specific CH_4_ yields ranged from ~48 to 112 mL CH_4_/g VS/day. For both parameters, decreasing the RS:DM ratio resulted in significantly lower specific yields (*p* < 0.001). Further, percent CH_4_ content within biogas from RS100 and RS90 were ~51 and 48% CH_4_, respectively, which are significantly higher than in RS30 and RS70 (41 and 43% CH_4_, respectively).

In contrast to biogas results, RS30 had the highest mean VS% removals (i.e. 38%), significantly higher than the other three reactors, which ranged between 23 to 26% (*p* = 0.003). RS30 also had the lowest mean VS residuals (4.7 g VS/L), significantly lower than the other three reactors (range from 11 to 13 g VS/L; *p* < 0.001). Differences in pH among reactors also were significant. RS30 had the highest mean pH (6.3), although all four reactors had pHs > 6.0. Differences in total VFA levels among reactors also were apparent; i.e. RS30 VFA levels were significantly lower than the other three reactors (mean of 97 ppm), which ranged from 401 to 506 ppm (*p* < 0.003). Although various VFAs were measured throughout the experiment, individual acids were not always present; therefore, only formic, acetic, isobutyric and propionic acid had adequate data to be statistically compared. Of these, propionic acid in RS30 was significantly lower (37 ppm) than the other three acids, which were between 246 and 319 ppm (*p* < 0.003). Time course data (Fig. 1a–d) show performance parameters were generally consistent during the period when samples were collected for microbial community characterisation.

### Beta-diversity and physiochemical parameters

**Fig. 2:**
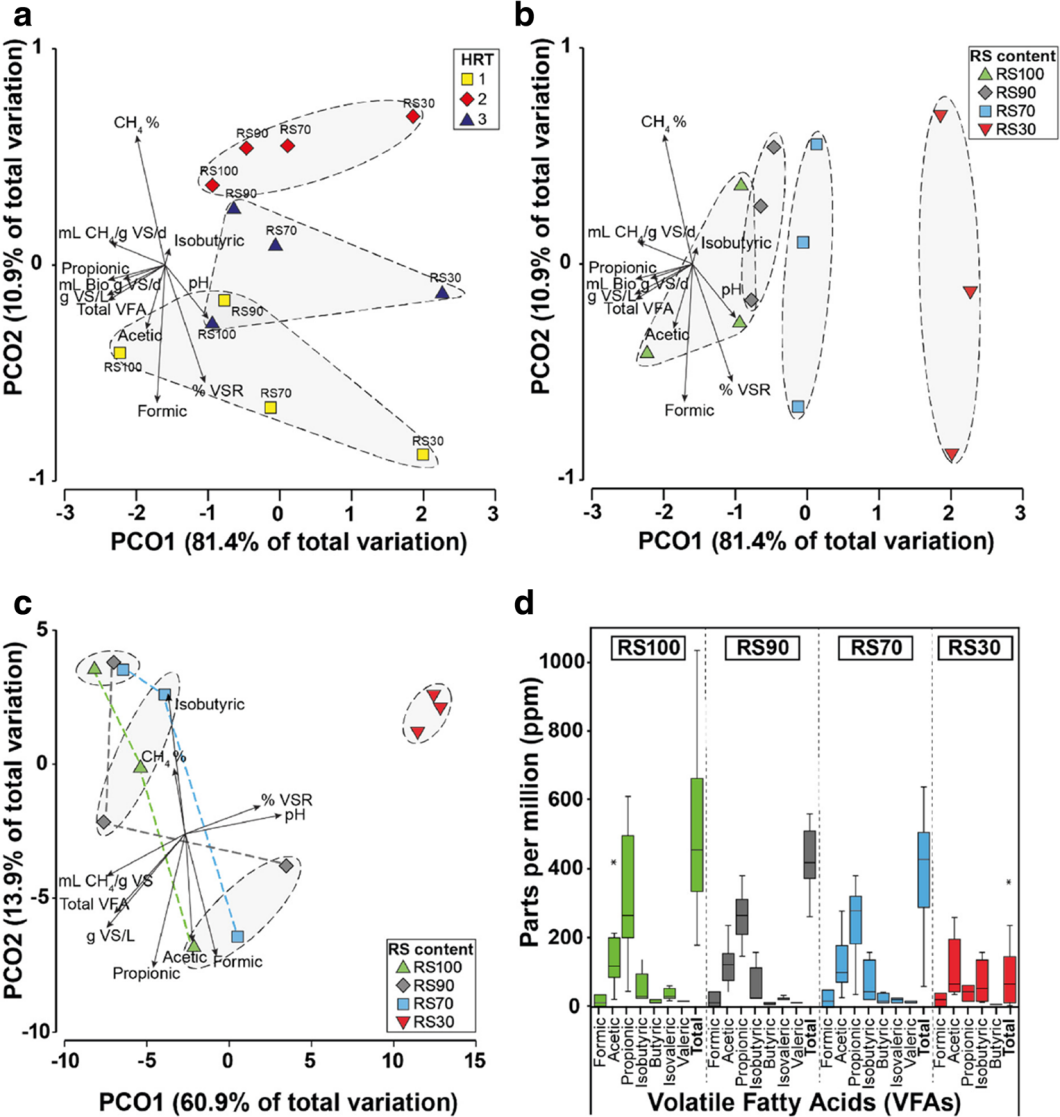
Analyses of beta diversity showing variation of microbial community structure and the influence of physiochemical data. **a**, **b** PCO of Bray-Curtis distance, but coloured differently by HRT and RS:DM. **c** PCO of weighted UniFrac distance. **d** Boxplot of individual and total VFAs for RS100, RS90, RS70 and RS30 (there was no isovaleric or valeric acid in RS30). Physiochemical data overlaid arrows and dashed elliptical shapes and/or coloured lines indicate sample groupings

To contrast the richness and evenness of microbial communities between reactors, beta-diversity indices were compared using ‘observed’ and Chao1 OTU numbers, and also Simpson’s and Shannon’s indices (Fig. 2). Based on Bray-Curtis distances, reactor samples clustered according to HRT (Fig. 2a) and RS:DM ratio (Fig. 2b), with RS:DM ratio having the greater effect with an axes variation of > 80%. The relative influence of each operating parameter is reflected by direction and length of the corresponding arrows. The weighted Unifrac distances (Fig. 2c) grouped the samples based on HRT (ellipses) and on RS:DM (shown by coloured lines), which shows RS30 groups separately from the other reactors in terms of reactor performance. For example, lower RS in the feed resulted in lower Total VFA levels, especially acetic, propionic and isobutyric acid (Fig. 2d).

To determine whether the groupings and correlations seen in Fig. 2 were statistically significant, RELATE, BEST, DistLM, ANOSIM and PERMANOVA were calculated (see Table 4). ANOSIM and PERMANOVA analysis showed that HRT and RS:DM are significantly different in terms of combined physiochemical data. However, ANOSIM and PERMANOVA showed that while RS:DM was significant in determining differences in microbial communities among reactors (i.e. beta-diversity; 2.2% at *p* = 0.018), HRT effects are apparent, albeit were not significant (7.3% at *p* = 0.276). The influence on beta-diversity of physiochemical operating conditions was shown by a significant 0.67 correlation (0.2%; RELATE) with pH (0.725), followed by %CH_4_, total VFAs, formic and isobutyric acid combined (0.724), providing the BEST correlations. DistLM analysis showed a range of variables were significantly related to beta-diversity, including biogas yield (*p* = 0.012), %CH_4_ (*p* = 0.039) and pH (*p* = 0.002).

**Table 4:** Test statistics for beta diversity as a function of physiochemical variables and other operational factors

Method: Relate			
Variable		Significance (%)	Rho.
Physiochemical data		*0*.*2*%	0.677
Method: Best			
Variable		Physiochemical correlation (*R*)	
pH		0.725	
CH_4_%, Total VFAs, formic, and isobutyric		0.724	
Method: DistLM			
Variable		*p* value	Cumulative variance explained (%)
Marginal			
mL Biogas/g VS/day		*0*.*012*	–
CH_4_%		*0*.*039*	–
mL CH_4_/g VS/day		*0*.*006*	–
g VS/L		*0*.*004*	–
% VSR		*0*.*021*	–
pH		*0*.*003*	–
Total VFA		*0*.*005*	–
Sequential			
+ pH		*0*.*002*	49.4%
Method: ANOSIM			
	Factor	Global R	Significance level (%)
Physiochemical	RS:DM	1.0	*0*.*3*
	HRT	0.75	*4*.*7*
Beta-diversity	RS:DM	0.81	*2*.*2*
	HRT	0.667	7.3
Method: Permanova			
	Factor	*p* value	Sq.root of estimates of component of variation
Physiochemical	RS:DM	*0*.*002*	1.49
	HRT	*0*.*004*	0.57
Beta-diversity	RS:DM	*0*.*018*	2.62
	HRT	0.276	7.26

Tests–RELATE, giving correlation of comparisons (Rho); BEST, trend correlation; DistLM, distance based linear model; ANOSIM, analysis of similarities; PERMANOVA, permutational multivariate analysis of variance

^a^Italic indicates statistically significant results

### Impact of co-digestion on alpha-diversity

**Fig. 3:**
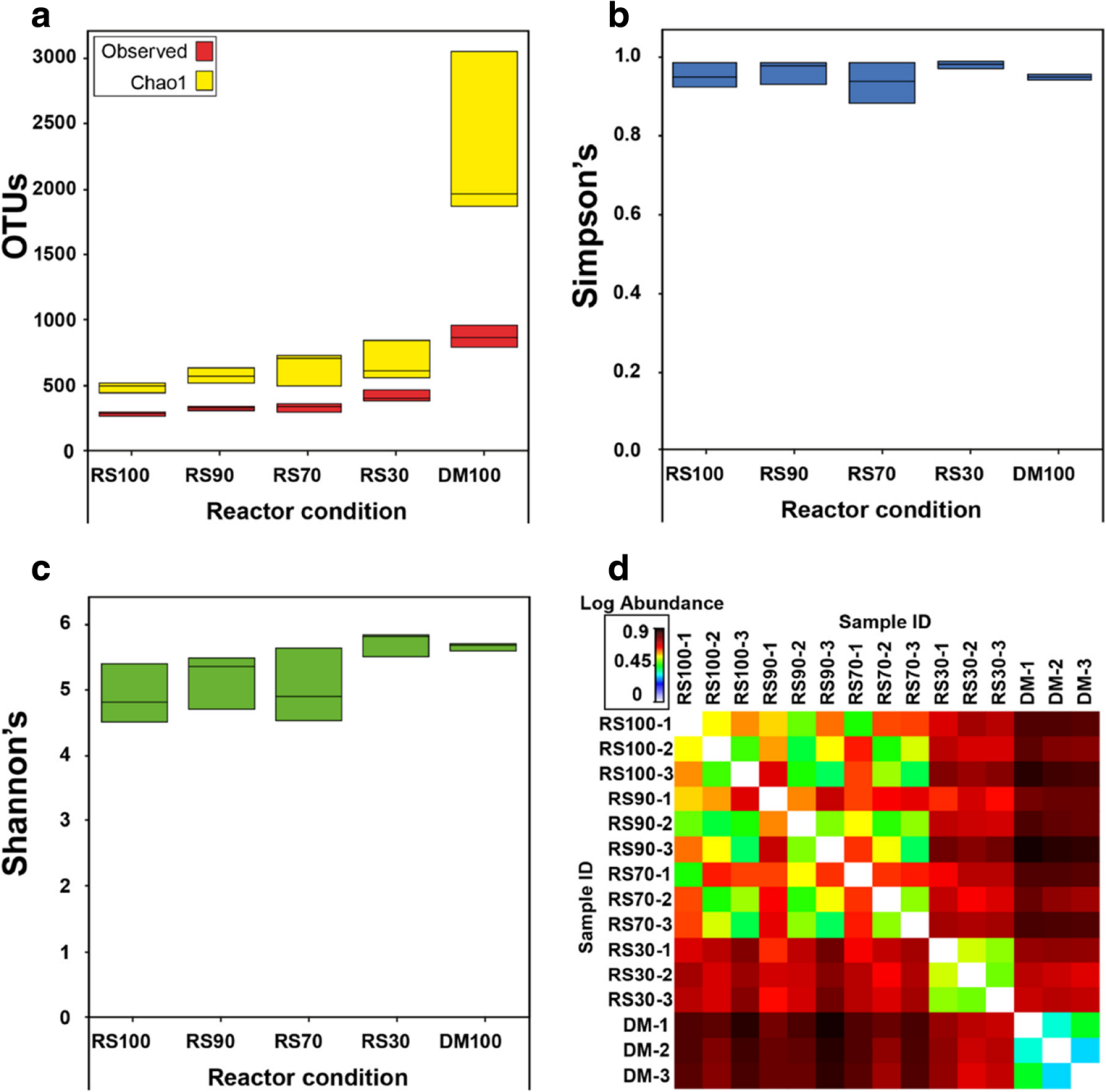
Boxplots of **a** Observed OTUs (lower) and Chao1 (upper boxes). **b** Simpson’s index scores. **c** Shannon’s index scores and **d** Heat map of beta-diversity abundances

Alpha-diversity indices (Hughes et al. 2001; Lemos et al. 2011) were used to contrast the richness and evenness of microbial communities in the reactors, based on observed and Chao1 OTU numbers (given as Figure S2), and Simpson’s and Shannon’s indices (Fig. 3). Observed OTUs and Chao1 estimations show that raw manure has significantly higher numbers of OTUs compared with any reactor communities (*p* < 0.001) followed by RS30, RS70 and RS90, although only RS100 had significantly lower OTUs than RS30 (*p* = 0.041). Chao1 estimations also showed that raw DM had significantly higher OTUs than any reactor (*p* < 0.001), which means it has a higher number of OTUs only sequenced once. Both these estimates show that as DM addition decreases, reactor community richness decreases (Fig. 3d), although Simpson’s and Shannon’s scores show differences were not always statistically significant (*p* > 0.05). Shannon’s score considers both richness and evenness, and it was higher for RS30 and DM (5.6 and 5.5). All diversity scores observed here, even in the reactor without DM addition, were quite high (Simpson’s = 0.90–0.95; Shannon’s = 4.8–5.6), especially compared with Zhao et al. (2012).

### Predominant OTUs

**Fig. 4:**
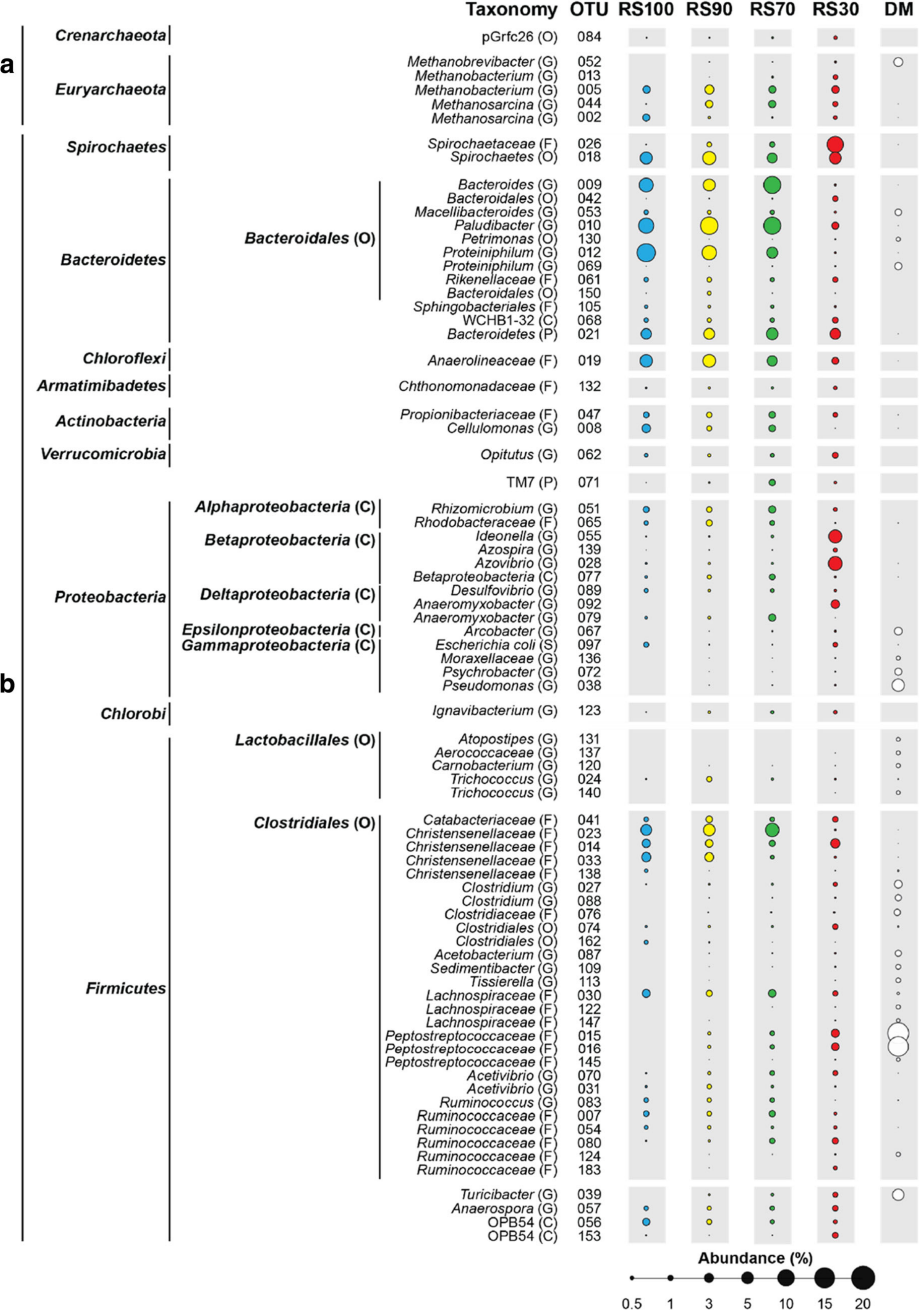
Predominant OTUs (≥ 0.5% abundance) to genus level where possible for RS100, RS90, RS70, RS30 and DM. A = archaea, B = bacteria. Area of bubbles represents relative abundance

**Fig. 5:**
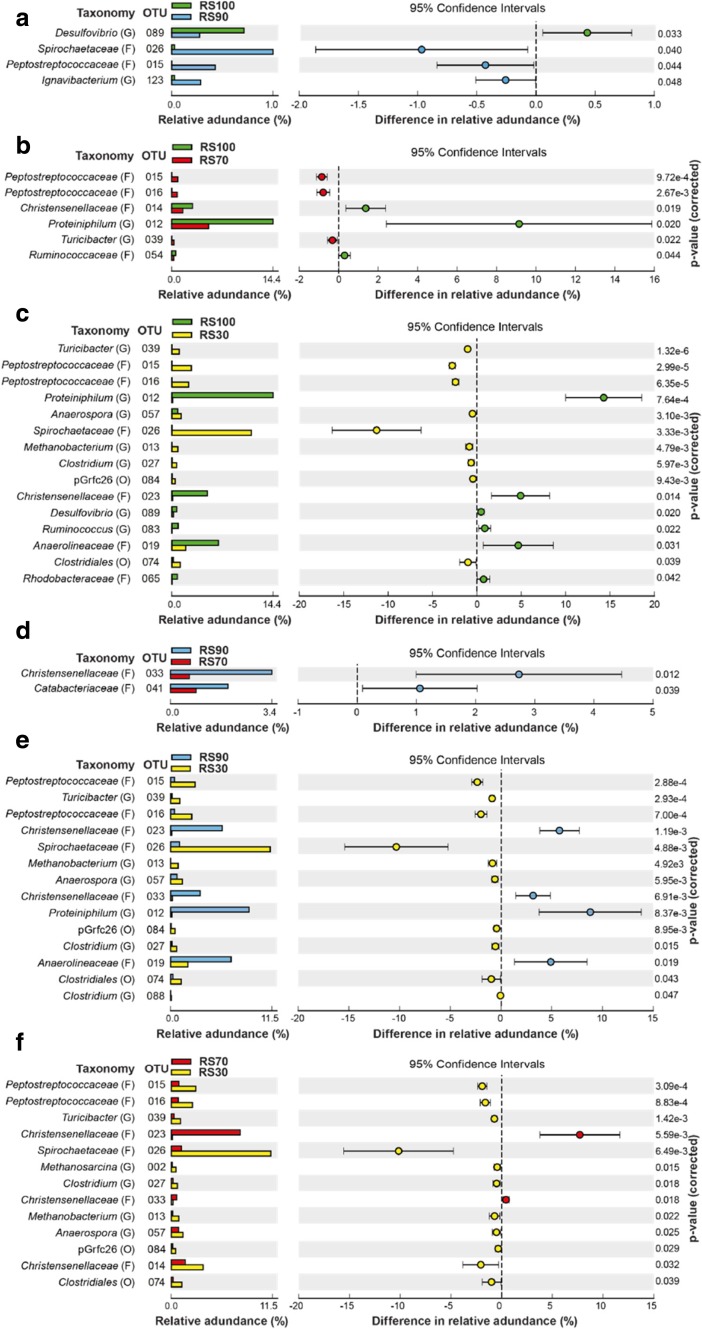
Extended error bar plot of significant differences between predominant OTUs. **a** RS100 vs RS90, **b** RS100 vs RS70, **c** RS100 vs RS30, **d** RS90 vs RS70, **e** RS90 vs RS30 and **f** RS70 vs RS30

To assess predominant OTUs, sequencing data were combined for all samples from each reactor because operational data over time did not significantly differ. Predominant OTUs are summarised in Fig. 4 (≥ 0.5% relative abundance, 76 OTUs and ‘others’ that is the cumulative abundance of those > 0.1%), which contrasts differences in major OTUs among reactors. The 76 major OTUs across reactors (not including the cumulative ‘others’) are provided as a phylogenetic tree in the SI (see Figure S3). Further, Fig. 5 shows if any perceived changes among conditions were statistically significant (all RS communities are compared with DM in Figures S4 to S7).

RS100, RS90 and RS70 were generally similar in phyla and dominated by *Firmicutes* and *Bacteroidetes* with the remaining 30–40% fraction made up of *Proteobacteria*, *Spirochaetes*, *Euryarchaeota* and *Chloroflexi*. RS30 differed in that *Bacteroidetes* was less prevalent and had higher levels of *Proteobacteria* and *Spirochaetes*. DM samples were > 65% *Firmicutes* and had almost no *Spirochaetes*. The number of predominant OTUs in each reactor was positively related with DM addition, ranging from RS100 and RS90 (32 and 31 OTUs, respectively) through RS70 (35 OTUs) to RS30 (38 OTUs). The DM samples also had a higher number of rare OTUs as their predominant OTU total was the lowest at 26.

Methanogens were present in all reactors, ranging from 4.5 to 6.2% in terms of relative abundance. Highest abundances were seen in RS90 and the lowest in RS100. *Methanobacterium* and *Methanosarcina* always were the main methanogen guilds, including OTUs identified as 005 and 044/002, with abundances ranging from 2.2 to 3.5 and 1.1 to 2.4%, respectively. Interestingly, these two *Archaea* taxa were below detection limits in DM samples. *Methanobrevibacter* (OTU 052) was dominant in the DM samples (3.4%), but was lower than 0.2% in all the reactors.

*Proteiniphilum* (OTU 012) was significantly more abundant in RS100 and RS90 (14.4 and 8.9%, respectively) than in RS30 (0.1% at *p* < 0.001 and 0.008). *Anaerolineaceae* abundance in RS100 and RS90 was 6.6 and 6.9%, lower in RS70 (4.4%), but significantly lower in RS30 (2.0% at *p* = 0.031 versus RS100 and 0.019 versus RS90). *Christensenellaceae* was significantly higher in RS90 (3.4% for OTU 033) than in RS30 (*p* = 0.007). *Ruminococcus* (OTU 083) generally had low abundance (~1%), but was significantly higher (*p* = 0.022) in RS100 (0.9% than RS30 (< 0.1%)). *Rhodobacteraceae* was highest in RS90 (1.6%) and lowest in RS30 (< 0.1%, *p* = 0.042) with 0.8 and 1.0% in RS100 and RS70. *Christensenellaceae* (OTU 023) was 5.0 and 5.9% in RS100 and RS90, but was highest in RS70 (7.8%) and lowest in RS30 (0.1%).

Relative abundance of *Spirochaetaceae* (OTU 026) was highest in RS30 (11.3%), which was significantly higher than in the other reactors, versus RS100 (> 0.1%), RS90 (1.0%) and RS70 (1.2%) at ≤ 0.006. *Clostridium* (OTU 027) and *Clostridiales* (OTU 074) in RS30 (0.7 and 1.2%) were significantly higher than in any other reactor (all *p* < 0.05), implying that higher DM addition might result in slightly higher putative pathogen levels than at lower DM additions. *Peptostreptococcaceae* (OTU 015 & 016) were both < 1.0% in RS100, RS90 and RS70, but were significantly higher in RS30 at 2.8 and 2.4% (all *p* ≤ 0.001).

## Discussion

### Effect of dairy manure addition on reactor performance

Specific biogas and CH_4_ yields were significantly greater at higher RS:DM ratios, although actual %CH_4_ content in the biogas was fairly similar across DM additions. In contrast, increasing DM additions resulted in slightly higher pH, greater VS% removals and lower levels of VS and VFA accumulation. Lower specific CH_4_ yields were unexpected as co-digestion with manure is touted as a method for enhancing RS AD processes (Marañón et al. 2012).

Based on RS stoichiometry, RS AD appears N-limited and N amendment as DM should improve nutritional balance within RS AD reactors (Wang et al. 2014). However, increasing DM additions significantly reduced specific CH_4_ yields in our AD reactors. This also was observed by Callaghan et al. (2002) and Dechrugsa et al. (2013) who found increasing levels of manure had a detrimental effect on biogas yields. This contradicts other RS co-digestion studies, such as Estevez et al. (2012), Wang et al. (2014), Sahito and Mahar (2014), Li et al. (2014a), Xavier et al. (2015) and Jiménez et al. (2016), who all found manure additions positively influenced biogas yields. However, none of these positive studies compared specific CH_4_ yields from RS side-by-side with and without manure addition; i.e. positive conclusions were made without suitable treatment controls. Adding DM to RS AD reactors reduced specific CH_4_ yields here relative to 100% RS reactors, although our OLRs were comparatively low.

However, DM addition did have positive benefits here that are reflected in non-methane AD performance data, which also was observed by Babaee et al. (2013). Manure addition almost certainly does balance the C:N ratio and provides additional nutrients to improve the AD process (Li et al. 2014a). Further, manure was shown by Li et al. (2015) and Cornell et al. (2012) to allow higher RS AD OLRs (both 6.0 kg VS/m^3^/day), which are six times higher than those used here. Therefore, at higher OLRs, the benefits of C:N balancing and extra nutrients may overweigh lower degradability of DM used for co-digestion. Therefore, we suspect the relative value of adding DM to RS AD systems depends on many factors, and specific CH_4_ yields with and without DM may differ dramatically depending on the specific RS and DM. To underpin results herein, 16S rDNA amplicon sequencing was performed to compare raw DM microbial communities with RS AD communities as a function of RS:DM in the reactor feed.

### Impact of DM addition on microbial communities in co-digestion systems

As RS content in the reactor feed was decreased from RS100 to RS30, reactor performance moved from higher biogas and VFA production to more stable pH and increased VS% removal in the systems (Fig. 2b). Further, greater accumulation of acetic, formic and propionic acids was evident (Fig. 2d) associated with greater CH_4_ production, which is consistent with higher RS in an AD unit generating higher gas yields and VFAs whilst lower RS leads to higher pH. In fact, ANOSIM and PERMANOVA show that RS:DM ratio was the dominant factor in determining reactor performance and also beta-diversity of the microbial community.

The addition of manure to RS AD reactors provides additional and varied microbes to the system (El-Mashad and Zhang 2010; Silvestre et al. 2013)*.* We see this here in richness and evenness data on alpha-diversity as indicated by Chao1, OTU numbers and Simpson and Shannon Indices. As DM level in the feed was increased, more diverse communities were apparent (Fig. 3), which is consistent with Mata-Alvarez et al. (2000) who made similar observations on the nature of meta-community.

Relative to specific OTUs, RS100, RS90 and RS70 all were similar in phyla and were dominated by *Firmicutes* and *Bacteroidetes*. In contrast, RS30 was dominated by *Proteobacteria* and *Spirochaetes*, and the raw DM samples were > 65% *Firmicutes* with almost no *Spirochaetes*. The lack of *Spirochaetes* in the DM is surprising given that Paster and Canale-Parola (1982) found *Spirochaetes* were important in the rumen, although in their case, they concluded *Spirochaetes* were less associated with cellulose processing than with the fermentation of plant polymers. Overall, RS AD microbial communities were significantly impacted by RS:DM ratio, especially when DM additions were high, but even with high DM addition, RS:DM were very different than DM itself.

*Methanobacterium* and *Methanosarcina* were the dominant methanogens in all our reactors, which also was found by Leite et al. (2015); Ziganshina et al. (2015) and Fontana et al. (2016). This dominance has been explained by the hydrogenotrophic traits of these guilds, allowing them to better cope with increases in hydrogen partial pressure (Goberna et al. 2010; Sun et al. 2015). Further, *Methanosarcina* dominance in RS100 also may be due to their substrate versatility, acid tolerance and higher specific growth rates (Conklin et al. 2006; Yi et al. 2014; Leite et al. 2015). The fact that *Methanosarcina* was apparently lower in the other reactors may be due to its limited acetoclastic abilities (FitzGerald et al. 2015); i.e. as acetic acid levels decreased, *Methanosarcina* also decreased (Fontana et al. 2016). Further, Leite et al. (2015) found that *Methanosarcina* preferred mono digestion rather than co-digestion environments, which is observed here.

No well-known syntrophic bacteria were observed in the reactors here. High hydrogen production by RS AD, as seen by Kim et al. (2012), potentially reduces the habitable zone for syntrophs that require low hydrogen pressures (Stams and Plugge 2009). A lack of syntrophs was unexpected when compared to other studies on sewage sludge digestion (Mei et al. 2017), although Liu et al. (2017) found that highly active fermenting bacteria could produce inhibiting levels of VFAs and hydrogen. Facultatively syntrophic bacteria, such as *Ruminococcus albus*, can grow syntrophically with hydrogenotrophic methanogens (Stams and Plugge 2009). However, the increase in acetic and propionic acid with increased RS suggests that syntrophic breakdown of acetate and propionate were overwhelmed (Amani et al. 2011; Banks et al. 2012). Deeper studies are required than those performed here to characterise obligate syntrophic guilds in RS AD, which could potentially further improve the efficiency of RS digestion.

There were a number of significant community changes across the reactors with some bacteria thriving in higher RS conditions. High abundance of *Bacteroidetes* then *Proteiniphilum* (OTU 012), which do not use cellulose (Chen and Dong 2005), was unexpected in the high RS reactors. However, these organisms can produce acetic and propionic acid (Krieg et al. 2015), which is consistent with higher VFA levels in RS100 and RS90. Among *Chloroflexi*, *Anaerolineaceae* (OTU 019) showed a similar pattern, being more abundant with a higher RS feed. Xia et al. (2016) found that the cellulolytic capacity of *Anaerolineaceae* was not likely to be its main attribute, although this observation is based on very limited data and its ecological role is uncertain. *Spirochaetaceae* (OTU 026) was higher in RS90 than RS100 and was significantly higher than in any other condition, although there are few cultivated species of this facultative anaerobe (Paster 2015). Of the four *Christensenellaceae* that can degrade cellulose (Fontes and Gilbert 2010), *Ruminococcus* (OTU 083) was in relatively low abundance, but was significantly higher in RS100 than RS30, which may be explained by its association with cellulose hydrolysis and its potential use of cellobiose (Sun et al. 2015).

Microbial communities in RS30 were quite different than the other three reactors. For example, *Firmicutes* phylum had the greatest number of increases in abundance in RS30 compared with RS100, although Lü et al. (2014) noted 16S rDNA sequence data on the *Firmicutes* phylum should be made with caution because of interferences and it is extremely complex. *Clostridium* (OTU 027) and *Clostridiales* (OTU 074) also were more evident in RS30, which is interesting given they are both higher in raw DM and are prospective pathogens. The presence of *Clostridium* and *Clostridiales* in RS30 may simply be legacy of higher DM additions, which may suggest that if DM is too high, strains in DM of potential health concern may prevail in AD digestates. *Peptostreptococcaceae* (family) (OTU 015 and 016) also were high in RS30 at 2.8 and 2.4%. Li et al. (2014b) found that *Peptostreptococcaceae* contains a number of genera isolated from manure and Mao et al. (2012) noted that it negatively correlates with VFAs, which may be why it was low in higher RS reactors. Regardless, most of the above genera may also be legacies of higher DM addition.

There were a number of ‘Goldilocks’ bacteria, favouring neither RS100 nor RS30, but something in the middle. *Rhodobacteraceae*, which is typical of cattle slurry (FitzGerald et al. 2015), was highest in RS90 (1.6%) and *Christensenellaceae* (OTU 023) was highest in RS70 (7.8%). There is currently only one described species of *Christensenellaceae*, which was found to favour gut environments (Morotomi et al. 2012; Rosa et al. 2017).

Overall, a greater number of predominant OTUs in reactors with greater DM addition implies that for RS:AD diverse OTUs are not essential for elevated specific CH_4_ yields. Clearly, greater diversity is related to lower VFA and VS accumulation associated with higher DM additions, although this is not due to greater biomass in higher DM reactors (see Table 3). In fact, increasing levels of DM addition progressively reduced biomass, although diversity increased. Although it is speculation, it is possible that DM additions introduce many diverse species that are not specifically related to methane production from RS, but more related to intermediate fermentation reactions. Therefore, higher DM addition appears to provide greater diversity among species responsible for VFA reduction, but not methanogenesis, which may be more specialised (i.e., *Methanobrevibacter* dominated DM samples, whereas *Methanosarcina* and *Methanobacterium* dominated the RS AD reactors). Overall, this implies co-digestion AD systems with higher DM might cope better at higher OLRs due to greater fermentation versatility, although Zealand et al. (2017) also showed that this capacity is not unlimited because higher OLRs can lead to reactor souring in RS AD units.

In conclusion, the highest specific CH_4_ yields were observed in the bioreactor without DM addition, whereas lowest yields were observed in the reactor with greatest DM additions. In contrast, as DM additions were increased, both VS and VFA accumulation decreased, and VS% removals increased. Further, increasing DM content in the feed resulted to greater microbial richness compared with reactors with higher levels of RS. Evenness was similar among RS:DM ratios, although the predominant OTUs differed among reactors. Higher RS AD reactors were dominated by *Firmicutes* and *Bacteroidetes*, whereas the reactor with the highest DM addition, RS30, was dominated by *Proteobacteria* and *Spirochaetes*, and also had detectable *Clostridium*, which may have implications to the subsequent use digestates as fertilisers.

Methanogen abundances were similar among the reactors, therefore lower abundances of cellulosic hydrolysing bacteria, such as *Christensenellaceae* and *Bacteroidetes*, best explain lower specific CH_4_ production levels when higher DM was in the feed. This hints that carbon short-circuiting may be occurring in the reactor with highest DM additions. However, the overall main benefit of co-digestion with RS and DM appears to be decreased VFA production and higher rates of VS removal, which suggest co-digestion systems can potentially operate at higher OLRs. Conversely, results suggest RS AD without DM also is a viable option, although overall RS throughput in RS only AD systems might be lower due to the need to operate at lower OLRs.

## Electronic supplementary material

ESM 1 (PDF 1499 kb)
