# Supplementary material for: Microbial community composition and diversity in rice straw digestion bioreactors with and without dairy manure
Source: Appl Microbiol Biotechnol. 2018 Jul 27;102(19):8599–612. doi: 10.1007/s00253-018-9243-7 (PMC6153884; doi:10.1007/s00253-018-9243-7)
Supplement: Supplementary file 1 — (PDF 1499 kb) [file 253_2018_9243_MOESM1_ESM.pdf]

Applied Microbiology and Biotechnology

Supplementary Information

Microbial Community Composition and Diversity in Rice Straw Co-digestion Bioreactors with Dairy Manure

A. M. Zealand[a], R. Mei[b], P. Papachristodoulou[a], A. P. Roskilly[c], W. T. Liu[b], and D. W. Graham[a*]

[a] *School of Engineering, Newcastle University, Newcastle upon Tyne, NE1 7RU, UK*

[b] *Department of Civil and Environmental Engineering, University of Illinois at Urbana-Champaign, 205 North Mathews Ave, Urbana, IL 61801, USA*

[c] *Sir Joseph Swan Centre for Energy Research, Newcastle University, Newcastle upon Tyne, NE1 7RU, UK*

*Corresponding author: Prof David W. Graham
School of Engineering
Cassie Building
Newcastle University
Newcastle upon Tyne
United Kingdom NE1 7RU
Phone: +44-191-208-7930
E-mail: david.graham@newcastle.ac.uk

Additional Methods in Next Generation Sequencing:

The PCRs included 1 to 10 ng of DNA extract (total volume 1µl), 15 pmol of each forward primer and reverse primer (in 20 µL volume of 1 x MyTaq buffer containing 1.5 units MyTaq DNA polymerase (Bioline) and 2 µl of BioStabII PCR Enhancer (Sigma). For each sample, the forward and reverse primers had the same 10-nt barcode sequence. PCRs were carried out for 30 cycles using the following parameters: 2 min 96°C pre-denaturation; 96°C for 15 s, 50°C for 30 s, 70°C for 90 s. DNA concentration of amplicons of interest was determined by gel electrophoresis. About 20 ng of amplicon DNA from each sample were pooled for up to 48 samples carrying different barcodes. If needed, PCRs with low yields were further amplified for 5 cycles.

The amplicon pools were purified with one volume AMPure XP beads (Agencourt) to remove primer dimer and other small mispriming products, followed by an additional purification on MinElute columns (Qiagen). About 100 ng of each purified amplicon pool DNA was used to construct Illumina libraries using the Ovation Rapid DR Multiplex System 1-96 (NuGEN). Illumina libraries were pooled and size selected by preparative gel electrophoresis. Sequencing was done on an Illumina MiSeq using V3 Chemistry (Illumina).

A total of 419,747 quality filtered samples were obtained from 15 samples with the number of 16S rDNA sequences ranging from 10,864 to 48,127 (mean, 27,983). After removing the chimera sequences the operational taxonomic units (OTUs) were picked at more than 97 % similarity representing 9,720 OTUs. The alpha and beta diversity of the microbial community were then assessed.

List of Figures:

Figure S1: Time-course data of digester performance including acclimation period (experiment 'Time 0' shown by dashed line) for pH, %Volatile Solids Removal (VSR), g VS/L, specific methane yields and VFA levels for: **a)** RS100, **b)** RS90, **c)** RS70, **d)** RS30.

Figure S2: Shared predominant OTU table to the genus level (only ≥ 0.5 % abundance) in bioreactor and raw dairy manure based on sample appearances. "H1", "H2", and "H3" refer to each HRT cycle (25 days) the reactors were operated.

Figure S3: Phylogenetic tree of predominant OTUs (only ≥ 0.5 % abundance) shared across all samples. Branch closeness and small white, grey, and black circles on branch junctions indicate percentage of similarity (50 - 69 %, 70 - 89 %, and 90+ %).

Figure S4: Extended error bar plot of significant differences between predominant OTUs in the RS100 reactor versus raw dairy manure (DM).

Figure S5: Extended error bar plot of significant differences between predominant OTUs in the RS90 reactor versus raw dairy manure (DM).

Figure S6: Extended error bar plot of significant differences between predominant OTUs in the RS70 reactor versus raw dairy manure (DM).

Figure S7: Extended error bar plot of significant differences between predominant OTUs in the RS30 reactor versus raw dairy manure (DM).

Figure S1

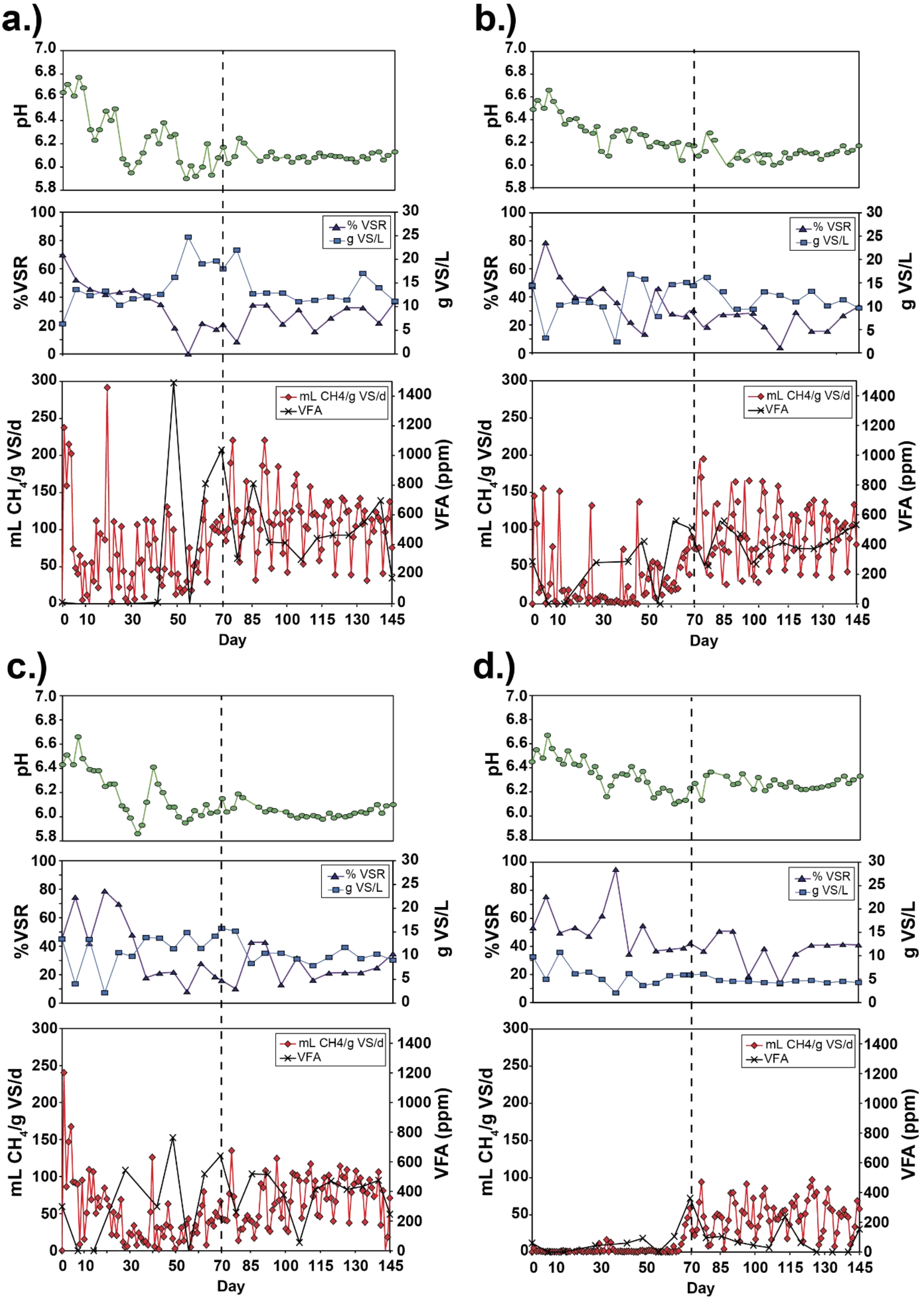
a.)
b.)
c.)
d.)
pH
%VSR
g VS/L
% VSR
g VS/L
mL CH4/g VS/d
VFA
mL CH$_4$/g VS/d
VFA (ppm)
Day

Figure S2

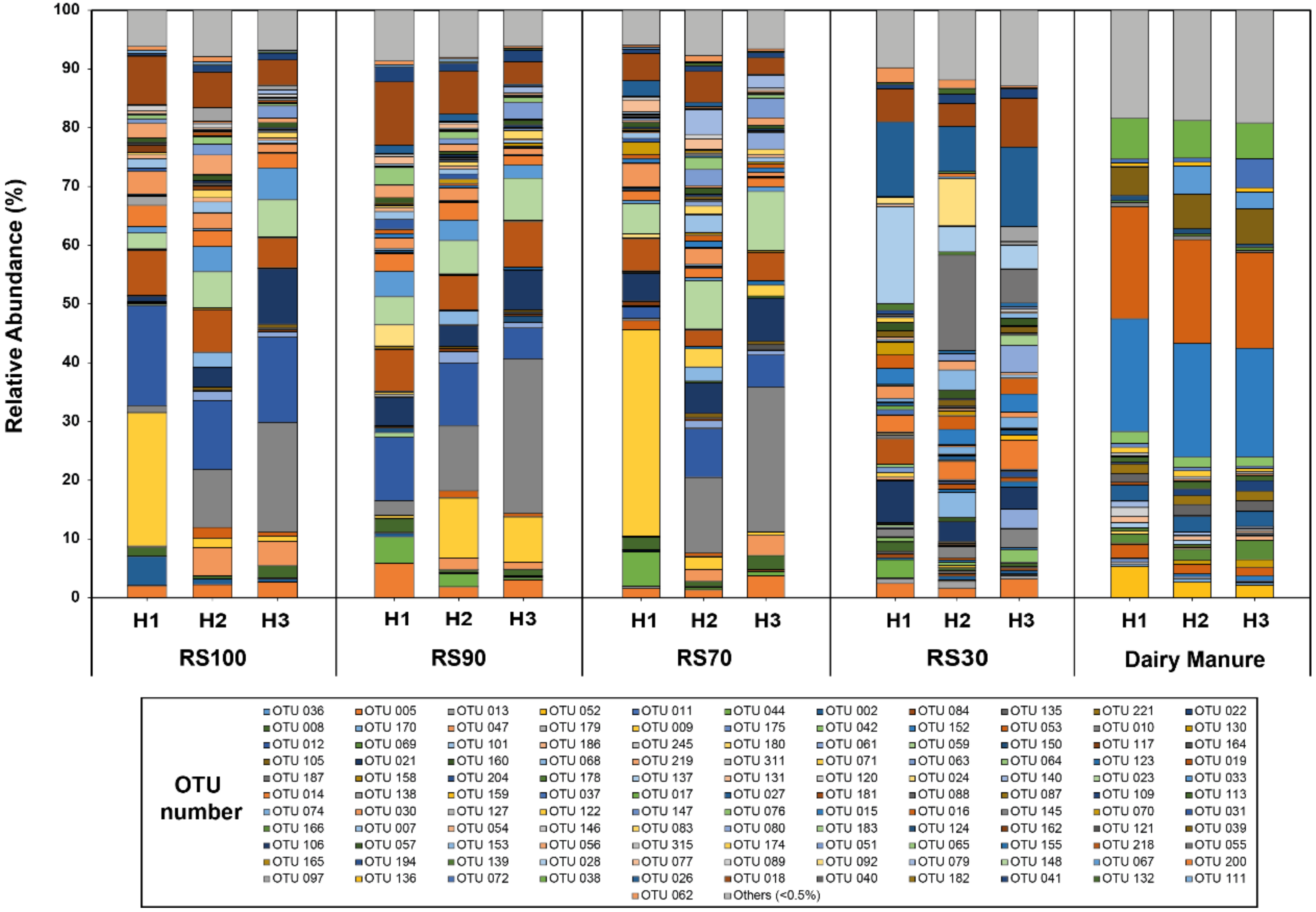
Relative Abundance (%)
100
90
80
70
60
50
40
30
20
10
0
H1
H2
H3
RS100
RS90
RS70
RS30
Dairy Manure
OTU number
OTU 036
OTU 005
OTU 013
OTU 052
OTU 011
OTU 044
OTU 002
OTU 084
OTU 135
OTU 221
OTU 022
OTU 008
OTU 170
OTU 047
OTU 179
OTU 009
OTU 175
OTU 042
OTU 152
OTU 053
OTU 010
OTU 130
OTU 012
OTU 069
OTU 101
OTU 186
OTU 245
OTU 180
OTU 061
OTU 059
OTU 150
OTU 117
OTU 164
OTU 105
OTU 021
OTU 160
OTU 068
OTU 219
OTU 311
OTU 071
OTU 063
OTU 064
OTU 123
OTU 019
OTU 187
OTU 158
OTU 204
OTU 178
OTU 137
OTU 131
OTU 120
OTU 024
OTU 140
OTU 023
OTU 033
OTU 014
OTU 138
OTU 159
OTU 037
OTU 017
OTU 027
OTU 181
OTU 088
OTU 087
OTU 109
OTU 113
OTU 074
OTU 030
OTU 127
OTU 122
OTU 147
OTU 076
OTU 015
OTU 016
OTU 145
OTU 070
OTU 031
OTU 166
OTU 007
OTU 054
OTU 146
OTU 083
OTU 080
OTU 183
OTU 124
OTU 162
OTU 121
OTU 039
OTU 106
OTU 057
OTU 153
OTU 056
OTU 315
OTU 174
OTU 051
OTU 065
OTU 155
OTU 218
OTU 055
OTU 165
OTU 194
OTU 139
OTU 028
OTU 077
OTU 089
OTU 092
OTU 079
OTU 148
OTU 067
OTU 200
OTU 097
OTU 136
OTU 072
OTU 038
OTU 026
OTU 018
OTU 040
OTU 182
OTU 041
OTU 132
OTU 111
OTU 062
Others (<0.5%)

Figure S3

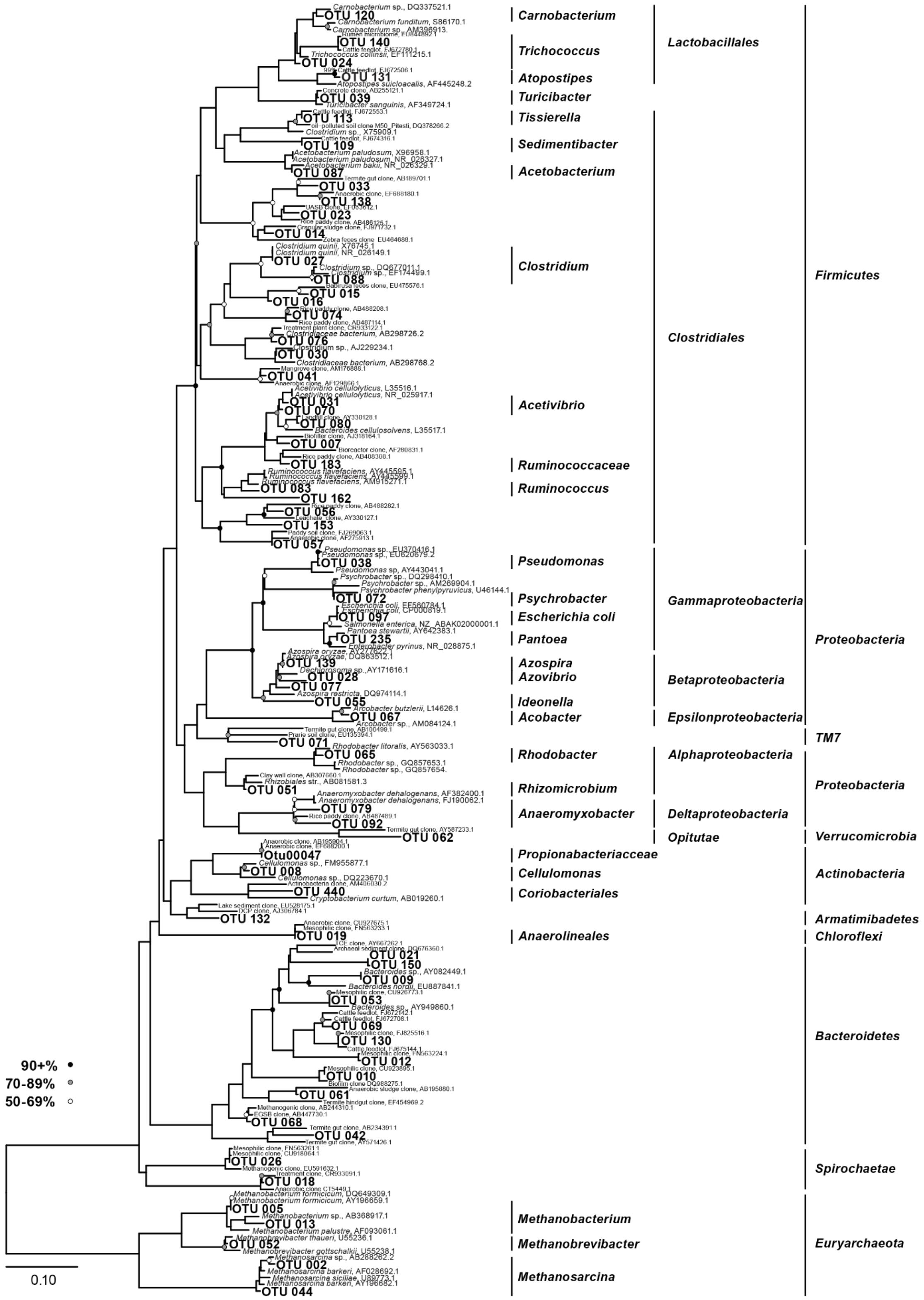

OTU 120
OTU 140
OTU 024
OTU 131
OTU 039
OTU 113
OTU 109
OTU 087
OTU 033
OTU 138
OTU 023
OTU 014
OTU 027
OTU 088
OTU 015
OTU 016
OTU 074
OTU 076
OTU 030
OTU 041
OTU 031
OTU 070
OTU 080
OTU 007
OTU 183
OTU 083
OTU 162
OTU 056
OTU 153
OTU 057
OTU 038
OTU 072
OTU 097
OTU 235
OTU 139
OTU 028
OTU 077
OTU 055
OTU 067
OTU 071
OTU 065
OTU 051
OTU 079
OTU 092
OTU 062
Otu00047
OTU 008
OTU 440
OTU 132
OTU 019
OTU 021
OTU 150
OTU 009
OTU 053
OTU 069
OTU 130
OTU 012
OTU 010
OTU 061
OTU 068
OTU 042
OTU 026
OTU 018
OTU 005
OTU 013
OTU 052
OTU 002
OTU 044
Carnobacterium
Trichococcus
Atopostipes
Turicibacter
Tissierella
Sedimentibacter
Acetobacterium
Clostridium
Acetivibrio
Ruminococcaceae
Ruminococcus
Pseudomonas
Psychrobacter
Escherichia coli
Pantoea
Azospira
Azovibrio
Ideonella
Acobacter
Rhodobacter
Rhizomicrobium
Anaeromyxobacter
Propionabacteriacceae
Cellulomonas
Coriobacteriales
Anaerolineales
Methanobacterium
Methanobrevibacter
Methanosarcina
Lactobacillales
Clostridiales
Gammaproteobacteria
Betaproteobacteria
Epsilonproteobacteria
Alphaproteobacteria
Deltaproteobacteria
Opitutae
Firmicutes
Proteobacteria
TM7
Proteobacteria
Verrucomicrobia
Actinobacteria
Armatimibadetes
Chloroflexi
Bacteroidetes
Spirochaetae
Euryarchaeota
90+%
70-89%
50-69%
0.10

Figure S4

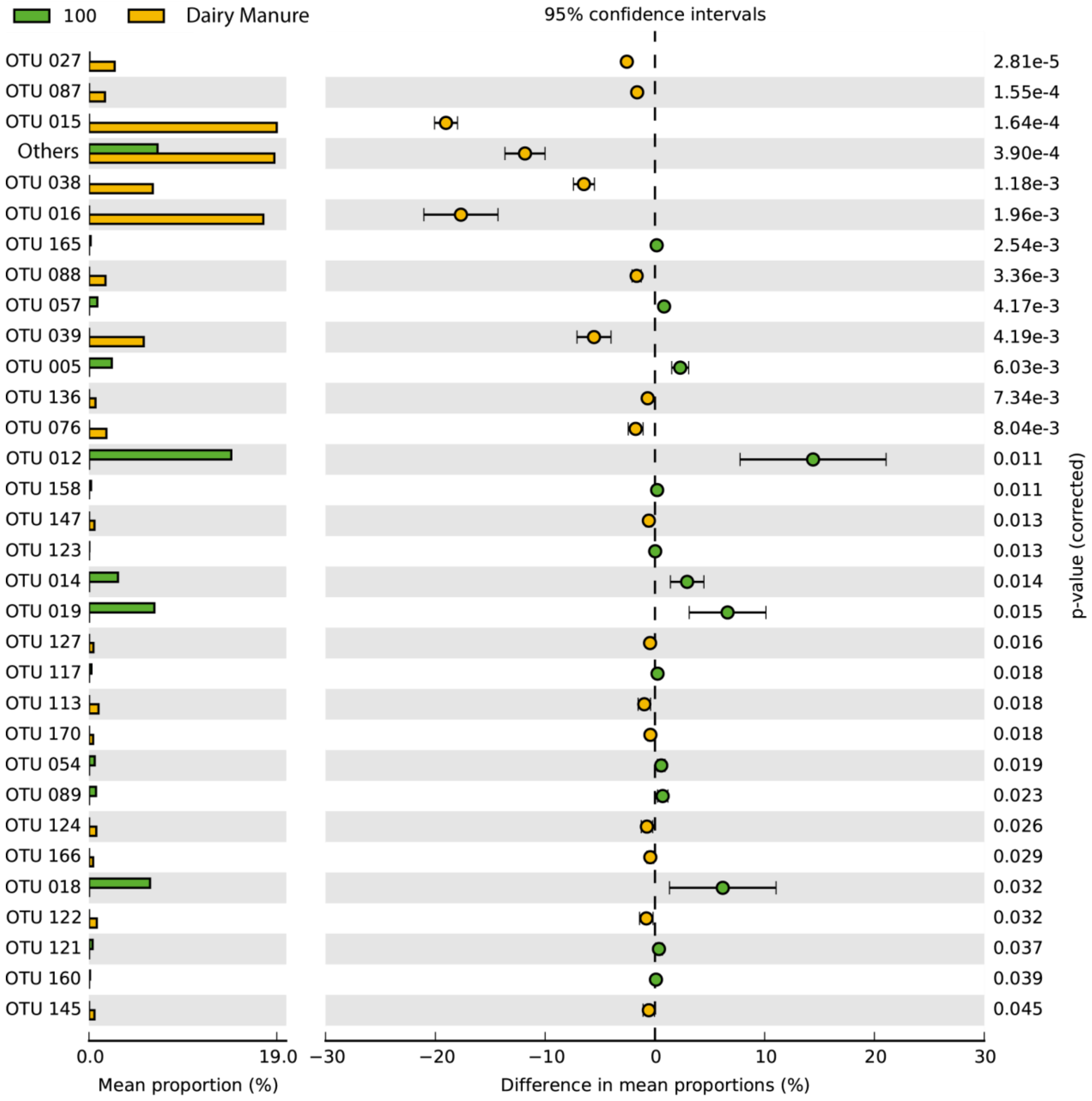
100
Dairy Manure
95% confidence intervals
OTU 027
OTU 087
OTU 015
Others
OTU 038
OTU 016
OTU 165
OTU 088
OTU 057
OTU 039
OTU 005
OTU 136
OTU 076
OTU 012
OTU 158
OTU 147
OTU 123
OTU 014
OTU 019
OTU 127
OTU 117
OTU 113
OTU 170
OTU 054
OTU 089
OTU 124
OTU 166
OTU 018
OTU 122
OTU 121
OTU 160
OTU 145
2.81e-5
1.55e-4
1.64e-4
3.90e-4
1.18e-3
1.96e-3
2.54e-3
3.36e-3
4.17e-3
4.19e-3
6.03e-3
7.34e-3
8.04e-3
0.011
0.011
0.013
0.013
0.014
0.015
0.016
0.018
0.018
0.018
0.019
0.023
0.026
0.029
0.032
0.032
0.037
0.039
0.045
p-value (corrected)
0.0
19.0
Mean proportion (%)
−30
−20
−10
0
10
20
30
Difference in mean proportions (%)

Figure S5

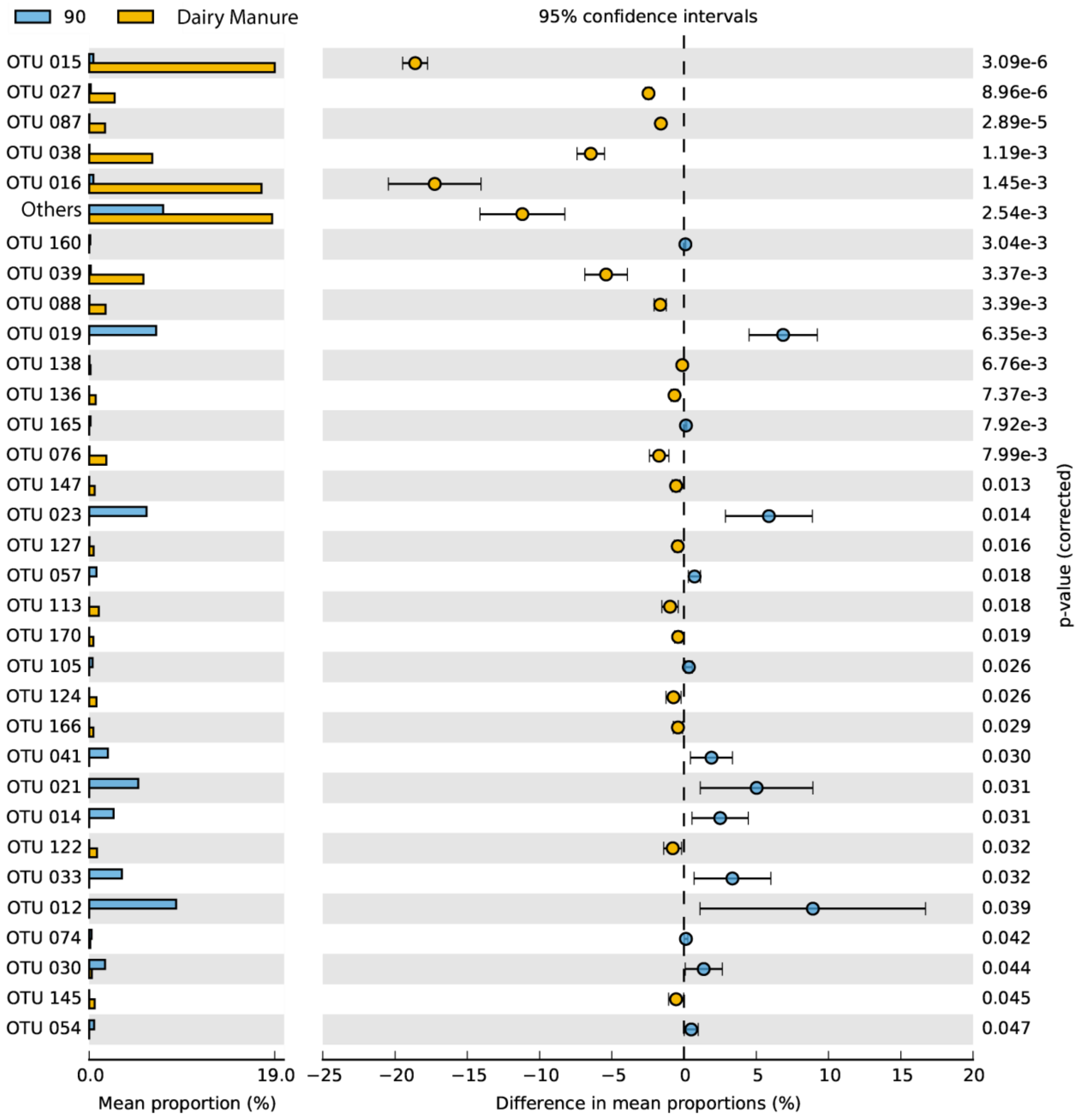
90
Dairy Manure
95% confidence intervals
OTU 015
OTU 027
OTU 087
OTU 038
OTU 016
Others
OTU 160
OTU 039
OTU 088
OTU 019
OTU 138
OTU 136
OTU 165
OTU 076
OTU 147
OTU 023
OTU 127
OTU 057
OTU 113
OTU 170
OTU 105
OTU 124
OTU 166
OTU 041
OTU 021
OTU 014
OTU 122
OTU 033
OTU 012
OTU 074
OTU 030
OTU 145
OTU 054
3.09e-6
8.96e-6
2.89e-5
1.19e-3
1.45e-3
2.54e-3
3.04e-3
3.37e-3
3.39e-3
6.35e-3
6.76e-3
7.37e-3
7.92e-3
7.99e-3
0.013
0.014
0.016
0.018
0.018
0.019
0.026
0.026
0.029
0.030
0.031
0.031
0.032
0.032
0.039
0.042
0.044
0.045
0.047
p-value (corrected)
0.0
19.0
Mean proportion (%)
−25
−20
−15
−10
−5
0
5
10
15
20
Difference in mean proportions (%)

Figure S6

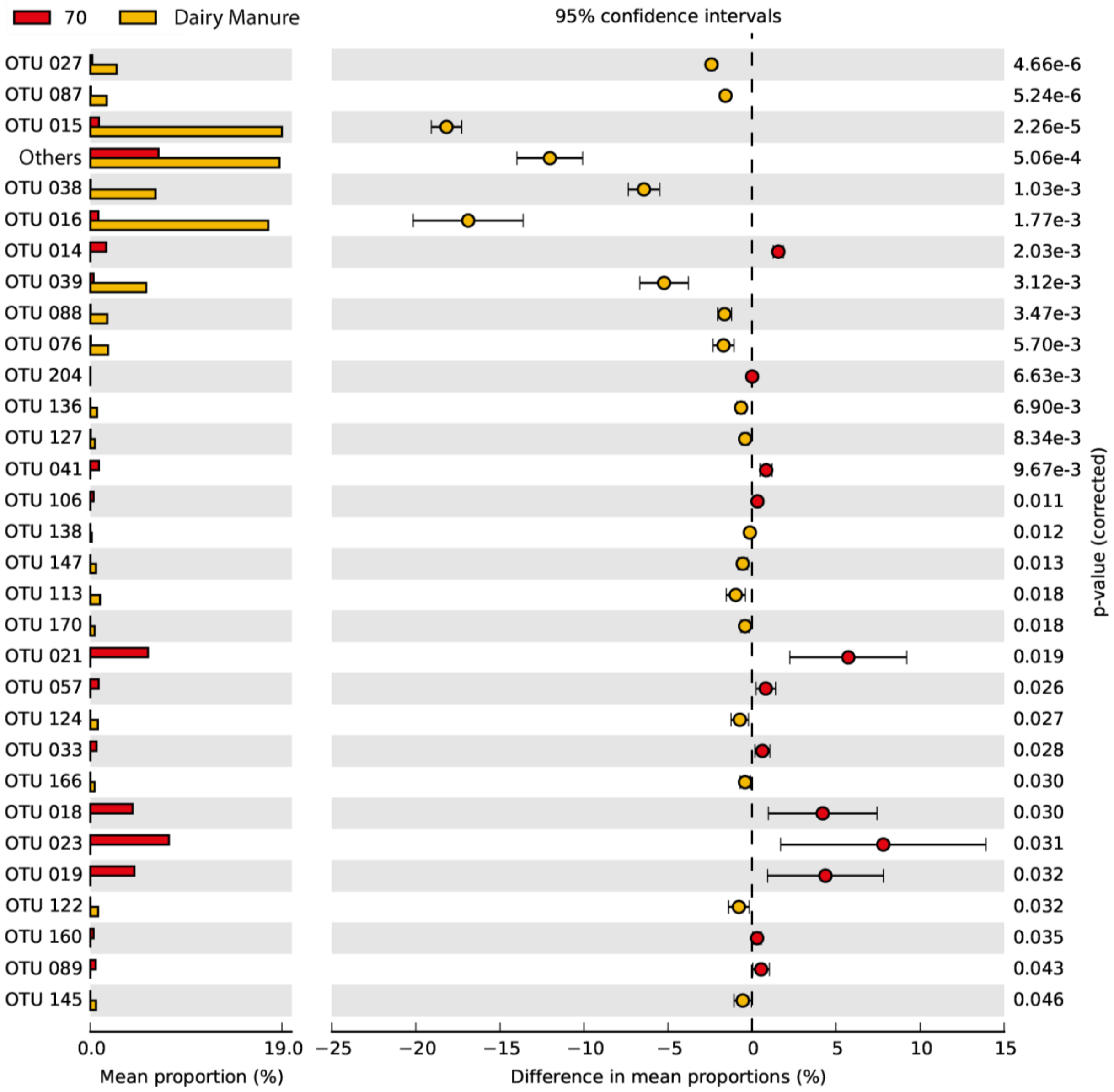
70
Dairy Manure
95% confidence intervals
OTU 027
OTU 087
OTU 015
Others
OTU 038
OTU 016
OTU 014
OTU 039
OTU 088
OTU 076
OTU 204
OTU 136
OTU 127
OTU 041
OTU 106
OTU 138
OTU 147
OTU 113
OTU 170
OTU 021
OTU 057
OTU 124
OTU 033
OTU 166
OTU 018
OTU 023
OTU 019
OTU 122
OTU 160
OTU 089
OTU 145
4.66e-6
5.24e-6
2.26e-5
5.06e-4
1.03e-3
1.77e-3
2.03e-3
3.12e-3
3.47e-3
5.70e-3
6.63e-3
6.90e-3
8.34e-3
9.67e-3
0.011
0.012
0.013
0.018
0.018
0.019
0.026
0.027
0.028
0.030
0.030
0.031
0.032
0.032
0.035
0.043
0.046
p-value (corrected)
0.0
19.0
Mean proportion (%)
−25
−20
−15
−10
−5
0
5
10
15
Difference in mean proportions (%)

Figure S7

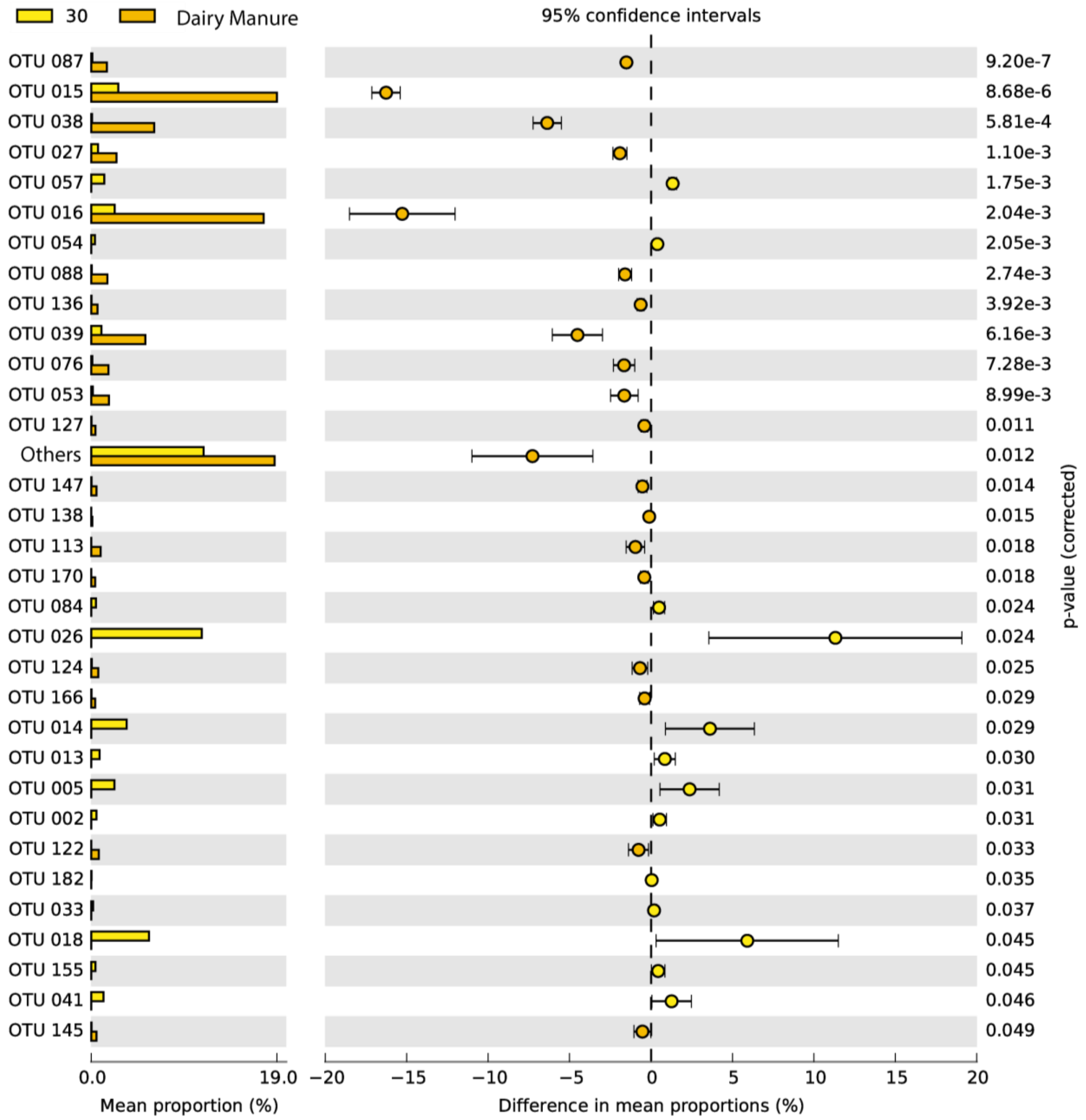

30
Dairy Manure
95% confidence intervals
OTU 087
OTU 015
OTU 038
OTU 027
OTU 057
OTU 016
OTU 054
OTU 088
OTU 136
OTU 039
OTU 076
OTU 053
OTU 127
Others
OTU 147
OTU 138
OTU 113
OTU 170
OTU 084
OTU 026
OTU 124
OTU 166
OTU 014
OTU 013
OTU 005
OTU 002
OTU 122
OTU 182
OTU 033
OTU 018
OTU 155
OTU 041
OTU 145
9.20e-7
8.68e-6
5.81e-4
1.10e-3
1.75e-3
2.04e-3
2.05e-3
2.74e-3
3.92e-3
6.16e-3
7.28e-3
8.99e-3
0.011
0.012
0.014
0.015
0.018
0.018
0.024
0.024
0.025
0.029
0.029
0.030
0.031
0.031
0.033
0.035
0.037
0.045
0.045
0.046
0.049
p-value (corrected)
0.0
19.0
Mean proportion (%)
−20
−15
−10
−5
0
5
10
15
20
Difference in mean proportions (%)
